# Correction: Kaur et al. Nanocomposite of MgFe_2_O_4_ and Mn_3_O_4_ as Polyphenol Oxidase Mimic for Sensing of Polyphenols. *Biosensors* 2022, *12*, 428

**DOI:** 10.3390/bios15070462

**Published:** 2025-07-18

**Authors:** Harmilan Kaur, Manpreet Kaur, Renuka Aggarwal, Sucheta Sharma, Davinder Singh

**Affiliations:** 1 Department of Chemistry, Punjab Agricultural University, Ludhiana 141004, India; harmilan2020-cm@pau.edu; 2 Department of Food and Nutrition, Punjab Agricultural University, Ludhiana 141004, India; renukaaggarwal@pau.edu; 3 Department of Biochemistry, Punjab Agricultural University, Ludhiana 141004, India; suchetasharma_pau@pau.edu; 4 Department of Extension Education, Punjab Agricultural University, Ludhiana 141004, India; davinder-ee@pau.edu

**Error in Figure/Table**

In the original publication [1], there was an overlay error while combining XRD spectra in Figure 1 and the calculated XRD parameters in Table 1. The corrected Figure 1, along with the XRD discussion and the recalculated XRD parameters in Table 1, are listed below. The authors state that the scientific conclusions are unaffected. This correction was approved by the Academic Editor. The original publication has also been updated.

**The XRD paragraph and Table 1 in Section 3.1.1 have been revised as follows:**

XRD was used to identify the phase and purity of synthesized NPs and nanocomposites. X-ray diffractograms displayed well-defined peaks (Figure 1), and the XRD parameters are listed in Table 1. The XRD pattern of H-1 NPs exhibited peaks at 2θ = 30.36°, 35.70°, 43.25°, 53.74°, 57.27°, 62.80°, 71.27°, and 74.38° [32,33]. These planes associated with the peaks proved to be strong evidence of the successful synthesis of MgFe_2_O_4_ (ASTM Data card No.17-465). Moreover, the absence of the extra peaks in the pattern made it evident that MgFe_2_O_4_ NPs were in a pure phase. The diffraction pattern of H-2 NPs showed sharp peaks at 2θ = 18.03°, 28.96°, 32.38°, 36.11°, 44.42°, 50.82°, 53.77°, 58.55°, 59.98°, and 64.67°, confirming the synthesis of Mn_3_O_4_ NPs (JCPDS Card No.24-0734). The Scherrer formula was used to recalculate the crystallite size (Table 1) of MgFe_2_O_4_ and Mn_3_O_4_, and their corrected values were 32 nm and 15 nm, respectively. The sharp diffraction peaks revealed that the NPs were well crystalline in nature. The combined peaks of H-1 and H-2 NPs were seen in the XRD patterns of H-3 and H-4, thus confirming their presence in the nanocomposite.

## Figures and Tables

**Figure 1:**
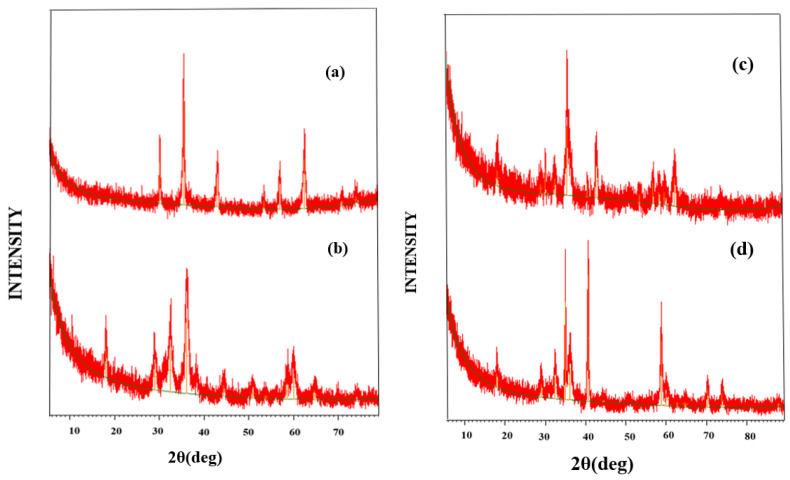
X-ray diffraction patterns of (**a**) (H-1), (**b**) (H-2), (**c**) (H-3), and (**d**) (H-4) nanoparticles.

**Table 1:** XRD parameters of nanoparticles.

Material Abbreviation (NPs)	(NPs)	Lattice Constant (Å)	Average Particle Diameter (nm)	XRD Density g/cc
H-1	MgFe_2_O_4_	8.36	32 nm	4.5
H-2	Mn_3_O_4_	a = 5.75 c = 9.57	15 nm	9.6
