# Supplementary material for: Nanocomposite of MgFe2O4 and Mn3O4 as Polyphenol Oxidase Mimic for Sensing of Polyphenols
Source: Biosensors (Basel). 2022 Jun 17;12(6):428. doi: 10.3390/bios12060428 (PMC9221326; doi:10.3390/bios12060428)
Supplement: Supplementary file 1 [file biosensors-12-00428-s001.zip › biosensors-1756428-supplementary.pdf]

biosensors

MDPI

Supplementary Data

Nanocomposite of $MgFe_2O_4$ and Mn_3O_4 as Polyphenol Oxidase Mimic for Sensing of Polyphenols

Harmilan Kaur [1], Manpreet Kaur [1,*], Renuka Aggarwal [2], Sucheta Sharma [3] and Davinder Singh [4]

[1] Department of Chemistry, Punjab Agricultural University, Ludhiana 141004, India; harmilan2020-cm@pau.edu
[2] Department of Food and Nutrition, Punjab Agricultural University, Ludhiana 141004, India; renukaaggarwal@pau.edu
[3] Department of Biochemistry, Punjab Agricultural University, Ludhiana 141004, India; suchetasharma_pau@pau.edu
[4] Department of Extension Education, Punjab Agricultural University, Ludhiana 141004, India; davinder-ee@pau.edu
* Correspondence: manpreetchem@pau.edu

Table S1. Details of characterization techniques.

S.No.	Instruments	Model
1.	Fourier Transformed Infrared spectroscopy (FT-IR)	Thermo Nicolet 6700 Fourier Transform Infrared Spectrometer
2.	Scanning electron microscopy with Energy dispersive spectroscopy (SEM-EDS)	Hitachi S-3400 and EDS on Thermo Noran System
3.	Transmission electron microscopy (TEM)	Hitachi Hi-7650 at 100 kV accelerate voltages in HC mode
4.	UV–Visible spectrophotometer	Shimadzu (UV-1800) UV spectrophotometer
5.	Vibrating sample magnetometer (VSM)	Model PAR-155
6.	X-ray Diffraction (XRD)	CuK_α radiations (λ = 1.5404 A^o) with a PanalyticalX pert Pro

Table S2. Variables, level of design experiments, and Box-Behnken Design (for catechol and resorcinol).

Factor	Value	Low	High
**pH**	A	1	9
**Temp (°C)**	B	10	60
**Catalytic Dose (mg)**	C	1	6
**Contact Time (min)**	D	2	20

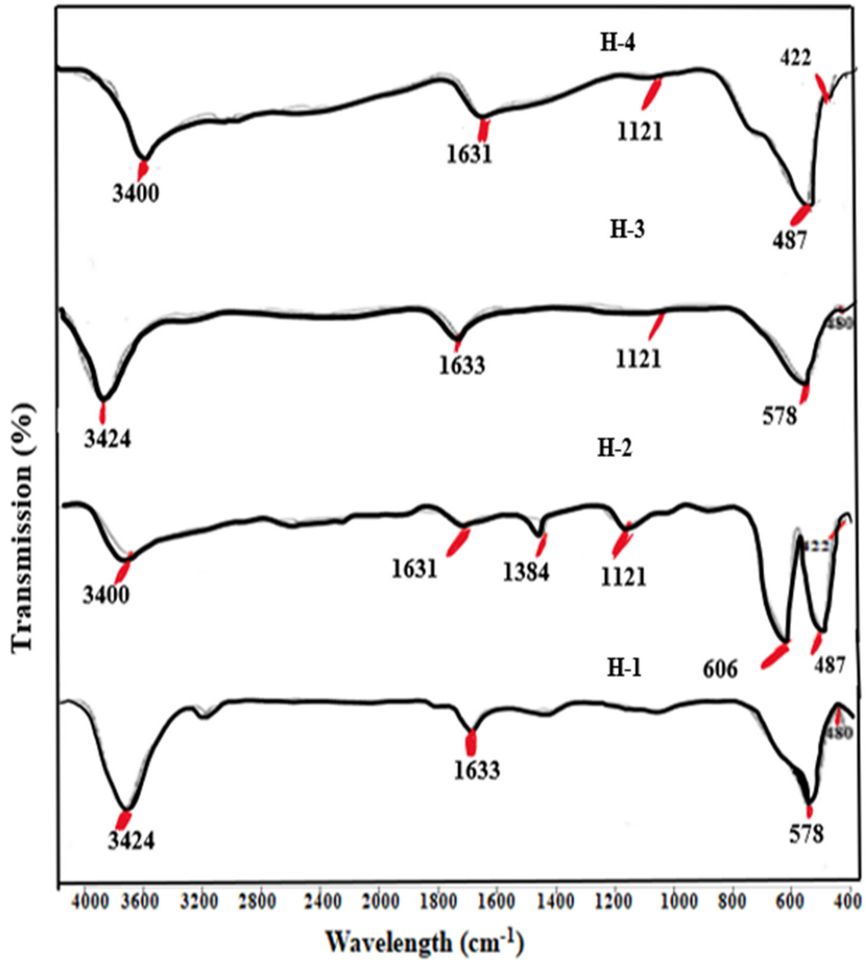

Figure S1. FT-IR spectra of H -1, H -2, H -3 and H -4 NPs.

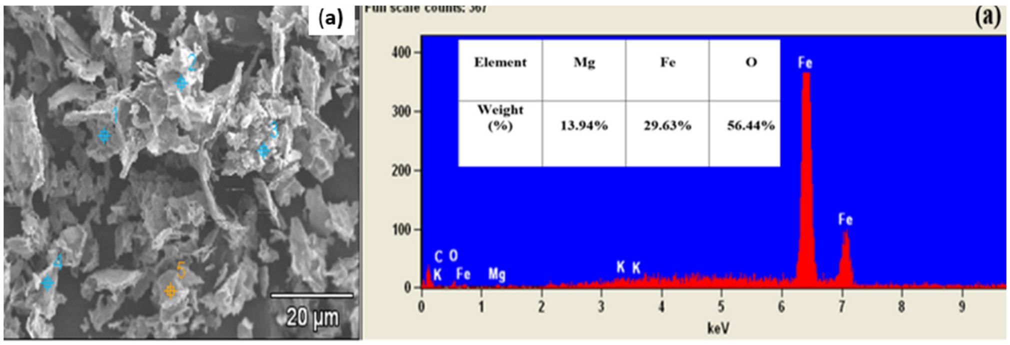

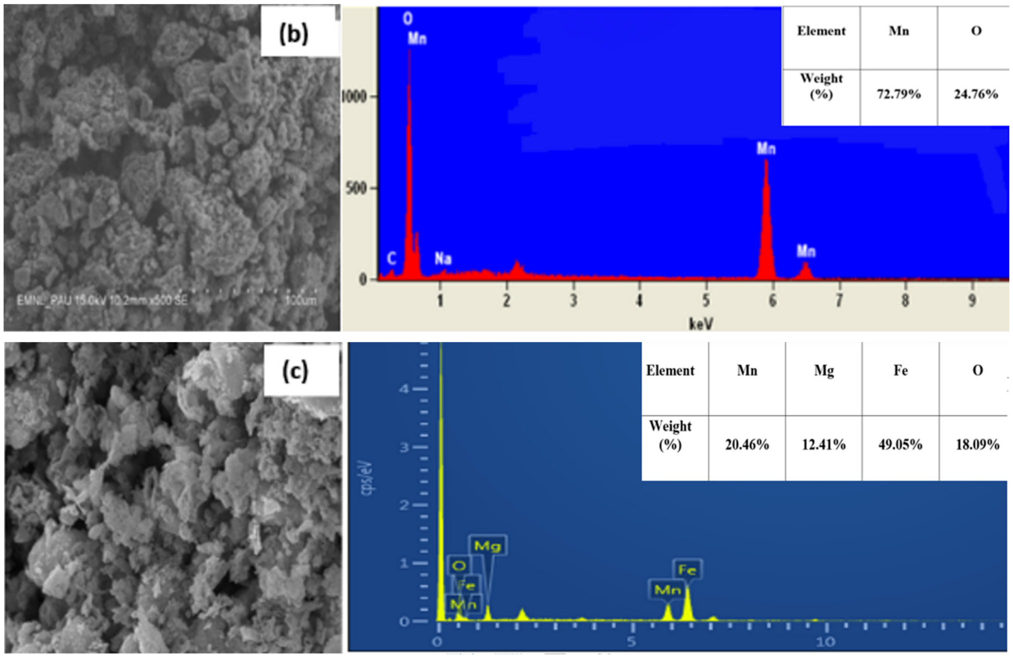

Figure S2. SEM EDS of (a) H-1 NPs (b) H-2 NPs (c) H-3 NC.

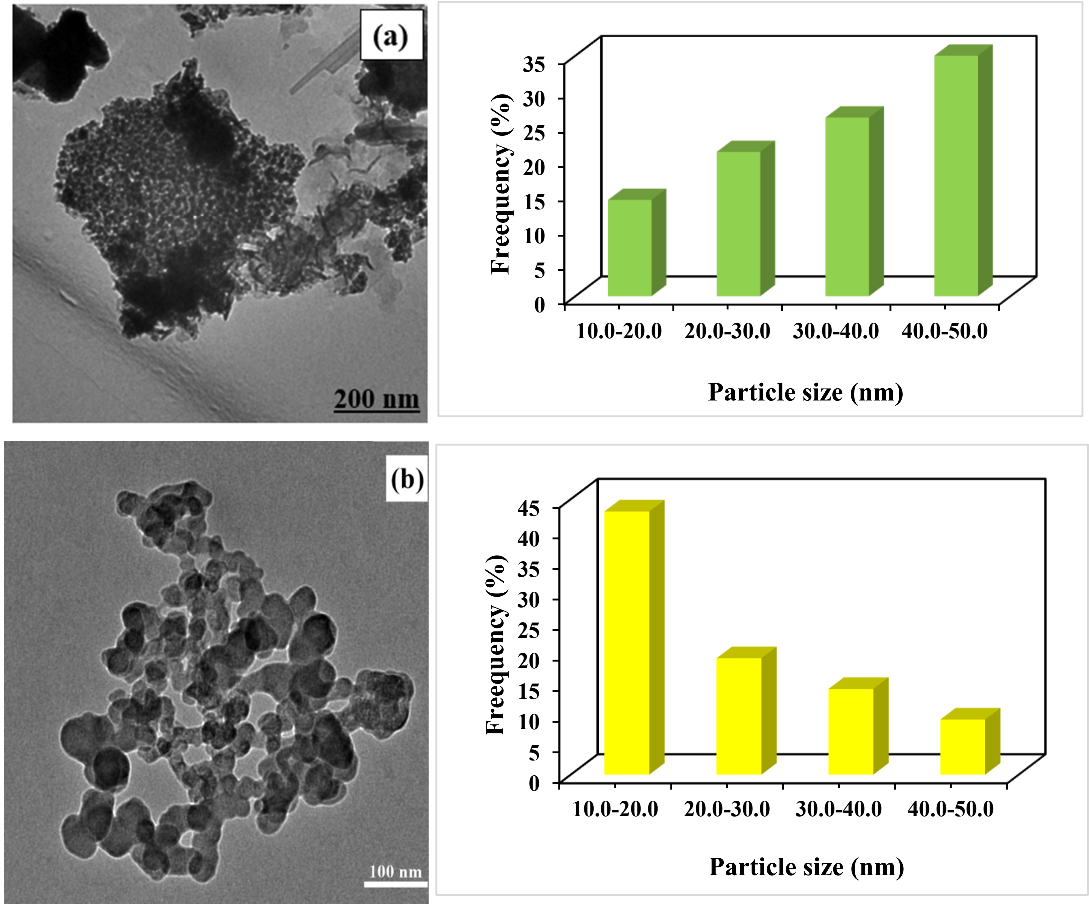

Figure S3. TEM of (a) H-1 NPs (b) H-2 NPs.

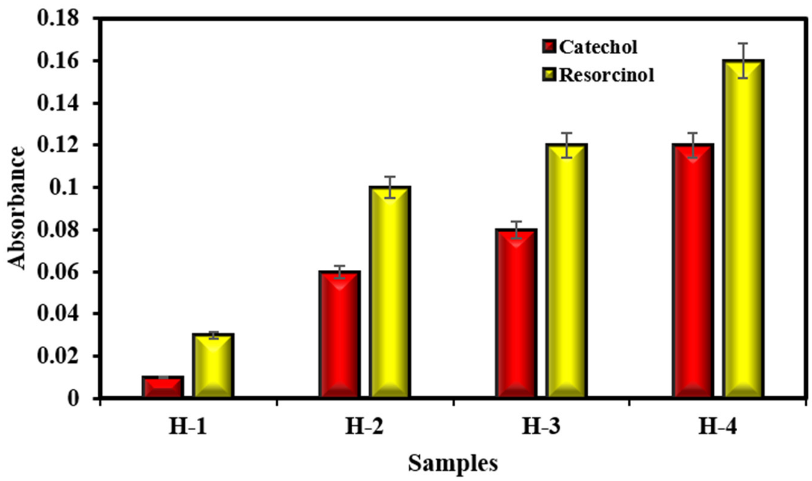

Figure S4. Comparison of activity of different samples.

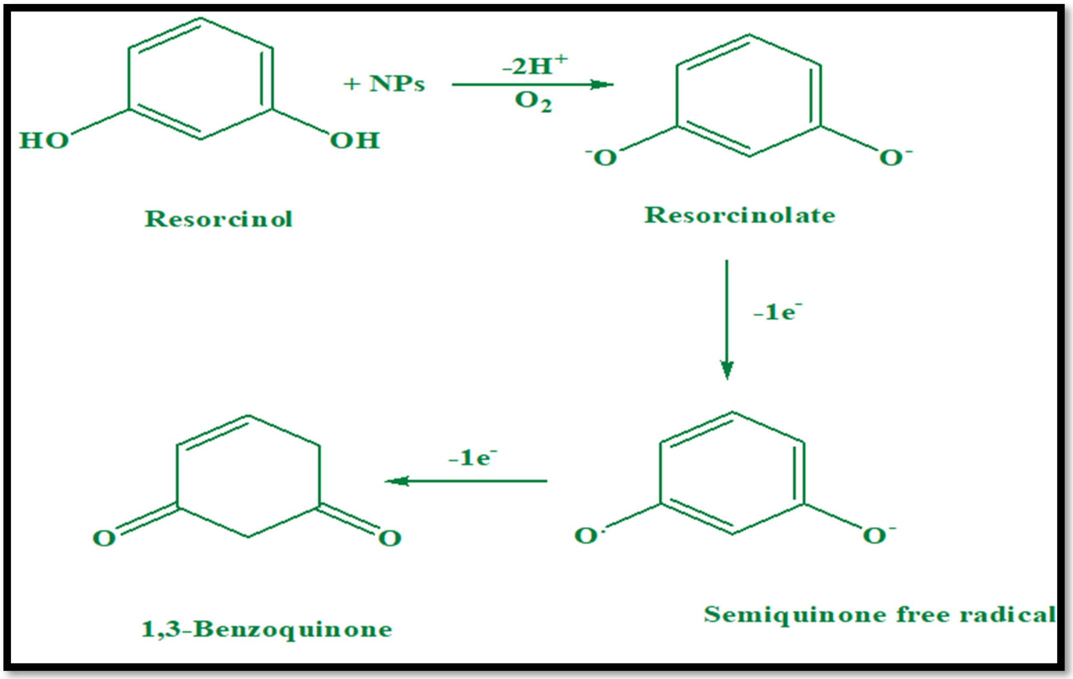

Scheme S1. Mechanism of PPO activity using resorcinol.

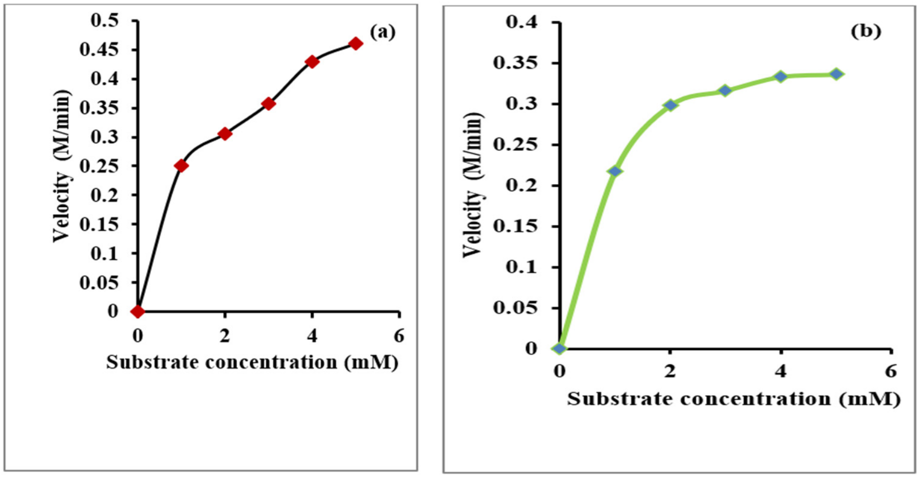

Figure S5. Plot of initial rates versus substrate concentration of oxidation catalysed by H-4 nanocomposite (a) Catechol (b) Resorcinol.

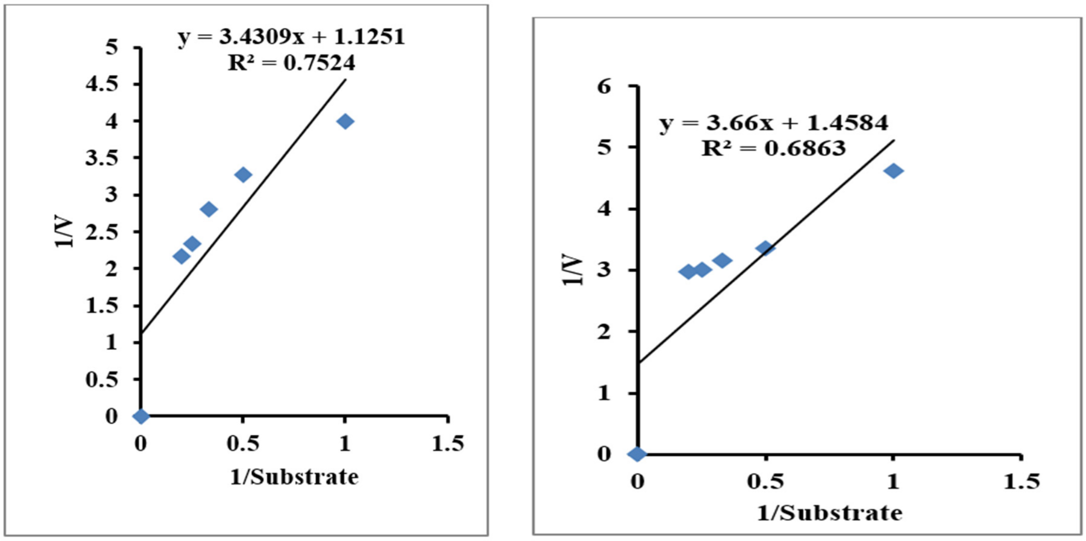

Figure S6. The lineweaver burk plot for H-4 NC (a) Catechol (b) Resorcinol.

Table S3. Kinetic parameters for PPO-like activity mention in text discuss.

Enzyme mimic	K_m (mM)	V_{max} (mol $L^{-1}min^{-1}$)	Reference
Mn_3O_4	1.14	0.52	Present work
H-4 NPs	0.71	0.336	Present work
Au@Pt NPs	1.29 x 10^2	13.97	[55]
$Fe(L^1)^2]SCN$	1.3	9.1 × 10^{-6}	[56]

Table S4. Analyses of variance for quadratic model of catechol.

Source	Sum of Squares	df	Mean Square	F-value	p-value	
Model	2.77	11	0.2518	4.04	0.0044	Significant
A-pH	0.1633	1	0.1633	2.62	0.1230	
B-Temp	0.0033	1	0.0033	0.0535	0.8198	
C-Catalytic Dose	0.0556	1	0.0556	0.5503	0.4686	
D-Contact Time	0.1633	1	0.1633	2.62	0.1230	

AB	0.0100	1	0.0100	0.1604	0.6935	
AD	1.0000	1	1.0000	16.04	0.0008	
BC	0.1225	1	0.1225	1.96	0.1781	
BD	0.3025	1	0.3025	4.85	0.0409	
CD	0.0025	1	0.0025	0.0401	0.8436	
C^2	0.4011	1	0.4011	6.43	0.0207	
D^2	0.4900	1	0.4900	7.86	0.0118	
Residual	1.12	18	0.0624			
Lack of Fit	0.8675	13	0.0667	1.31	0.4085	not significant
Pure Error	0.2550	5	0.0510			
**Cor Total**	3.89	29				

R^2 = 0.7116

Table S5. Analysis of variance for quadratic model of resorcinol.

Source	Sum of Squares	df	Mean Square	F-value	p-value	
**Model**	2.85	12	0.2375	2.45	0.0448	Significant
A-pH	0.1875	1	0.1875	1.93	0.1823	
B-Temp	0.0408	1	0.0408	0.4211	0.5250	
C-Catalytic Dose	0.0533	1	0.0533	0.5501	0.4684	
D-Contact Time	0.0133	1	0.0133	0.1375	0.7153	
AB	0.7225	1	0.7225	7.45	0.0143	
AC	0.2500	1	0.2500	2.58	0.1267	
BD	0.0225	1	0.0225	0.2321	0.6361	
CD	0.7225	1	0.7225	7.45	0.0143	
A^2	0.3344	1	0.3344	3.45	0.0807	
B^2	0.0744	1	0.0744	0.7674	0.3932	
C^2	0.2976	1	0.2976	3.07	0.0978	
D^2	0.1719	1	0.1719	1.77	0.2006	
**Residual**	1.65	17	0.0970			
Lack of Fit	1.56	12	0.1296	6.94	0.0219	Significant
Pure Error	0.0933	5	0.0187			
**Cor Total**	4.50	29				

R^2 = 0.6336

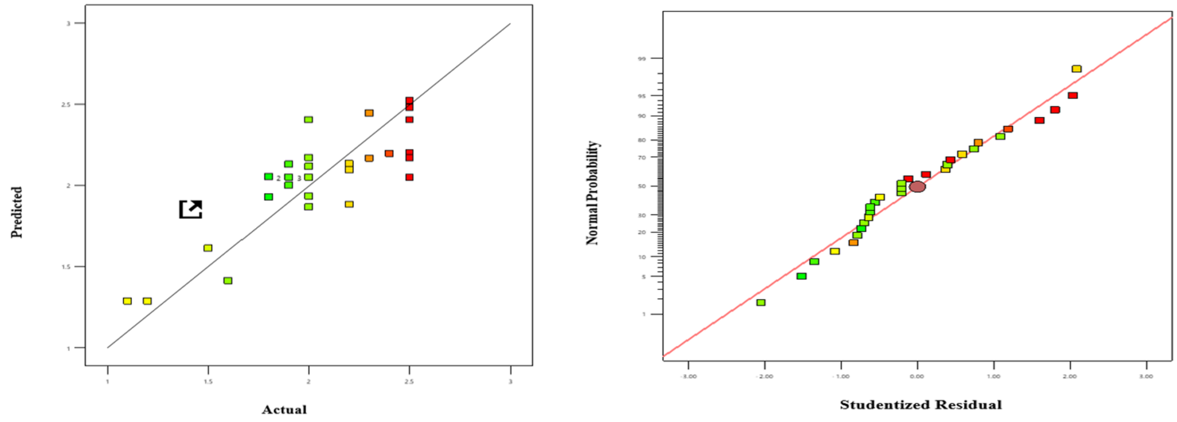

Figure S7. (a) Correlation between the experiments with predicted values of enzyme activity (using catechol), (b) Normal probability plot of residuals obtained by ANOVA for the enzyme activity of H-4 nanocomposite (using catechol).

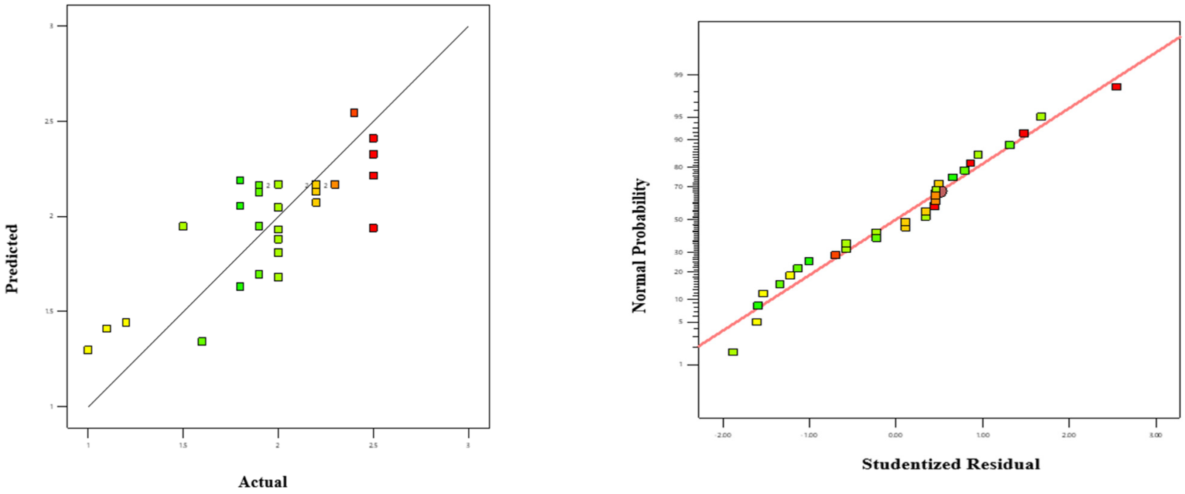

Figure S8. Correlation between the experiments with predicted values of enzyme activity (using resorcinol), (b) Normal probability plot of residuals obtained by ANOVA for the enzyme activity of H-4 nanocomposite (using resorcinol).

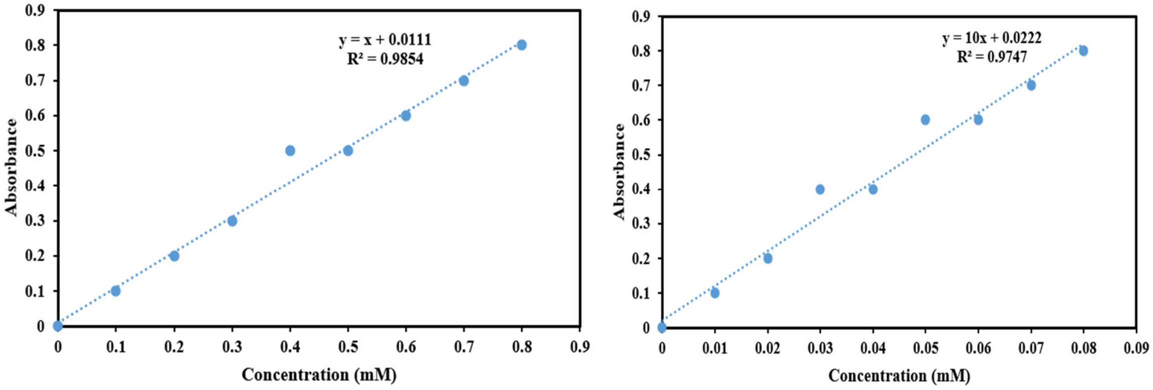

Figure S9. The linear caliberation plots for (a) catechol detection b) resorcinol detection.

(pH =3.0; Temp=25 °Cand 0.5 ml H_2O_2).

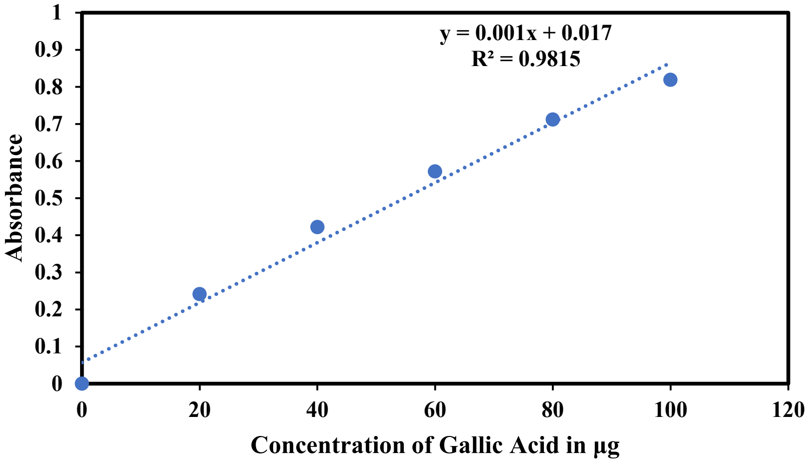

Figure S10. Caliberation plot of total phenolic content determined by the $MgFe_2O_4@Mn_3O_4$ nanocomposite based assay.
